# Using Expert Driven Machine Learning to Enhance Dynamic Metabolomics Data Analysis

**DOI:** 10.3390/metabo9030054

**Published:** 2019-03-20

**Authors:** Charlie Beirnaert, Laura Peeters, Pieter Meysman, Wout Bittremieux, Kenn Foubert, Deborah Custers, Anastasia Van der Auwera, Matthias Cuykx, Luc Pieters, Adrian Covaci, Kris Laukens

**Affiliations:** 1 Adrem Data Lab, Department of Mathematics and Computer Science, University of Antwerp, 2000 Antwerp, Belgium; Laura.peeters@uantwerpen.be (L.P.); pieter.meysman@uantwerpen.be (P.M.); wout.bittremieux@uantwerpen.be (W.B.); 2 Department of Genome Sciences, University of Washington, Seattle, WA 98195, USA; 3 Natural Products & Food Research and Analysis (NatuRA), Department of Pharmaceutical Sciences, University of Antwerp, 2000 Antwerp, Belgium; kenn.foubert@uantwerpen.be (K.F.); Deborah.custers@uantwerpen.be (D.C.); Deborah.custers@uantwerpen.be (A.V.d.A.); luc.pieters@uantwerpen.be (L.P.); 4 Toxicological Center, Department of Pharmaceutical Sciences, University of Antwerp, 2000 Antwerp, Belgium; matthias.cuykx@uantwerpen.be (M.C.); adrian.covaci@uantwerpen.be (A.C.)

**Keywords:** machine learning, dynamic metabolomics, data simulation

## Abstract

Data analysis for metabolomics is undergoing rapid progress thanks to the proliferation of novel tools and the standardization of existing workflows. As untargeted metabolomics datasets and experiments continue to increase in size and complexity, standardized workflows are often not sufficiently sophisticated. In addition, the ground truth for untargeted metabolomics experiments is intrinsically unknown and the performance of tools is difficult to evaluate. Here, the problem of dynamic multi-class metabolomics experiments was investigated using a simulated dataset with a known ground truth. This simulated dataset was used to evaluate the performance of tinderesting, a new and intuitive tool based on gathering expert knowledge to be used in machine learning. The results were compared to EDGE, a statistical method for time series data. This paper presents three novel outcomes. The first is a way to simulate dynamic metabolomics data with a known ground truth based on ordinary differential equations. This method is made available through the MetaboLouise R package. Second, the EDGE tool, originally developed for genomics data analysis, is highly performant in analyzing dynamic case vs. control metabolomics data. Third, the tinderesting method is introduced to analyse more complex dynamic metabolomics experiments. This tool consists of a Shiny app for collecting expert knowledge, which in turn is used to train a machine learning model to emulate the decision process of the expert. This approach does not replace traditional data analysis workflows for metabolomics, but can provide additional information, improved performance or easier interpretation of results. The advantage is that the tool is agnostic to the complexity of the experiment, and thus is easier to use in advanced setups. All code for the presented analysis, MetaboLouise and tinderesting are freely available.

## 1. Introduction

The field of metabolomics, which studies small molecules inside organisms, has expanded considerably over the last two decades and the amount of data being generated by metabolomics experiments keeps increasing. For many “typical” experiments, standardized workflows are available. Over the last years, many initiatives have focused on providing free and open access workflows and pipelines for metabolomics data analysis. For example, Workflow4Metabolomics [1] (W4M) provides an intuitive way of constructing workflows by linking modules together. These modules provide a multitude of steps for preprocessing, statistics, normalization and others. Many of these tools were originally written in R, python, etc. but have been converted to the Galaxy [2] environment that underlies W4M. The net result is that anyone has access to the most advanced tools, but more importantly the workflows can be automated, standardized and shared, thereby greatly improving verifiability and reproducibility of research and results. This push towards reproducibility is, for example, also apparent in the new release of MetaboAnalyst [3], which is accompanied by the R package MetaboAnalystR [4] to allow researchers to easily share their workflow code for identifying compounds.

For more complex experiments such as dynamic metabolomics, sometimes also called longitudinal or time-resolved metabolomics, standardized workflows are lacking. Small molecules and their processes are the direct result of biochemical activity and therefore, metabolomics describes an inherently dynamic process. There is a large variety of dynamic metabolomics experimental use cases, ranging from studying specific hormone levels in a single patient over a single day to find recurring patterns relating to biorhythm, to quantifying the effect of treatment for a certain known metabolite. An overview with examples and approaches to analyze these experiments can be found in the work by Smilde et al. [5]. This paper focuses on the data analysis of untargeted dynamic metabolomics experiments. We can illustrate the need for such experiments by considering the example of prodrug metabolism. Aspirin (acetylsalicylic acid), for example, is a prodrug that requires a bioconversion to become pharmacologically active [6]. In this process, certain metabolites are consumed and others are formed over a period of time. If it is unknown which biotransformation products are formed from possible active compounds or prodrugs, untargeted experiments can be used to identify these compounds. However, the data processing of such dynamic experiments is not trivial, as there are often multiple sample classes measured over multiple time points.

There is no common default strategy yet for the data analysis of large-scale, untargeted, dynamic metabolomics experiments, in which, simultaneously, many features and a large variety of different time profiles need to be considered. One promising solution mentioned by Smilde et al. [5] is EDGE [7,8]. This tool originates from the genomics field and uses natural cubic splines. Even though it is not widely used in metabolomics, it provides a promising solution for discriminating different time profiles. The drawback is that EDGE is specifically tailored to two-class problems. Using EDGE for multi-class experiments is not straightforward as problems arise both regarding the multiple testing issue and on the not trivial combination of individual two-class comparisons. Resolving this issue would take considerable time and expert knowledge as results need to be checked manually and compared to the raw data.

The workflow presented in this paper provides an easy and intuitive alternative that makes more efficient use of expert knowledge by incorporating it into the analysis pipeline. The knowledge gathering step is done in a playful manner that has been implemented in an app called “tinderesting”. Experts are iteratively shown figures of data entities, for example features, and rate each based on whether the feature is interesting or not. Next, this labeled data provided by the experts is used to train a machine learning model that in turn can be used to analyze the full dataset. The idea is in spirit analogous to the recaptcha concept, in which internet users are required to decipher jumbled-up words that are presented on the screen, assisting in elucidating hard to digitize text from old manuscripts [9].

In this paper, the tinderesting workflow is compared to the EDGE approach. The comparison is performed on simulated data to allow the comparison of results to the ground truth with quantifiable performance metrics such as receiver operator characteristics. To validate the approach on experimental data, Peeters et al. [10] used the tinderesting method to analyze experimental data, illustrating the usefulness of the approach in a real-world setting.

## 2. Materials and Methods

### 2.1. Simulating Dynamic Metabolomics Data

A data simulation method was developed to compare EDGE and tinderesting. The advantage of this approach is that the dataset can be constructed in such a way that the ground truth is known. However, constructing such a simulated longitudinal metabolomics dataset is not common practice. Therefore, a dedicated method is provided for simulating these data, based on a limited number of biologically inspired assumptions. These simulated data are visually comparable to actual dynamic metabolomics data [10].

The following biologically inspired assumptions were used to simulate the longitudinal metabolomics data:

(1) The dynamics in the data are governed by an underlying network with an appropriate connectivity distribution (the Barabási-Albert model was chosen [11,12], see Section S1 in Supplementary Materials for further discussion).
(2) Nodes in the network are metabolites, which have certain starting concentrations that evolve over time.
(3) Transitions in concentrations are caused by enzymes. Effectively, these are the rates/fluxes that govern flow in the network.
(4) Some of these enzymes are assigned to multiple edges and are also influenced by the adjacent nodes/metabolites. That way, an external influx in metabolite X can cause a depletion of metabolite Y somewhere else in the network (X increases, thus rate/flux of X to X’ increases, which is the same rate/flux as the one from Y to Y’, thus Y depletes).
(5) The intake of certain compounds (e.g., nutrients) causes an external influx in specific metabolites, (temporary rise in concentration).
(6) The rate/flux increases or decreases depending on the concentration of the metabolite causing the reaction. This increasing rate is limited and follows a sigmoidal curve.

**Figure 1:**
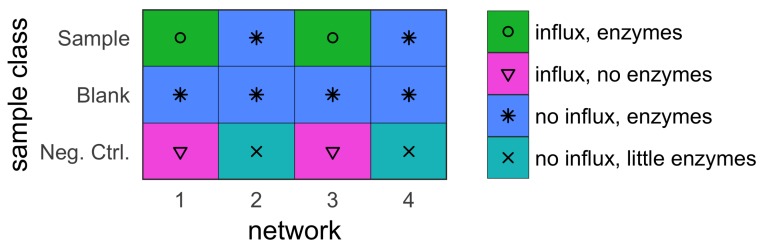
External tinderesting data, simulated setup.

**Figure 2:**
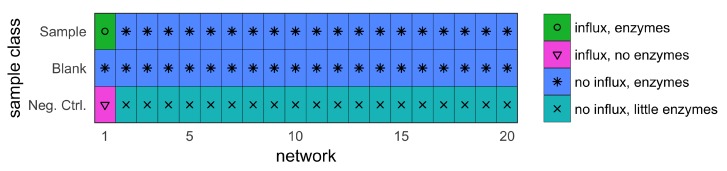
Visualized simulation setup. Only in the first network there are metabolites that differ between sample and blank as well as between sample and negative control. For the remaining 95% of metabolites, there is only a small difference in level of enzymes between sample and negative control. These enzymes convert one metabolite into the other with a rate dependent on the enzyme quantity. Networks 2–19 for the negative control case contain little enzymes instead because there are always some biotransformations occurring. Not including these would produce too large differences between sample and negative control. For the sample vs. blank case, the only difference is in the starting concentrations caused by the Gaussian noise term.

With these assumptions, two separate datasets were constructed. The first is a training dataset, which was used for the tinderesting approach. The second is a validation dataset, which was used to compare the machine learning model trained on the tinderesting results with the EDGE method. When using this method on experimental data, the training dataset would be a subset of the entire dataset (Peeters er al. [10]). Each of the datasets was constructed by combining the results of multiple smaller networks, each consisting of 50 metabolites (nodes). The training dataset consists of four of these 50-node networks to obtain a dataset with 200 metabolites in total (Figure 1). The validation dataset consists of twenty different networks to obtain a simulated dataset with 1000 metabolites (Figure 2).

Three different sample classes were simulated by adding different elements to the network. One or both of the following were added: enzymes to convert metabolites (rates between nodes) or external influxes. The different sample classes are differentiated by what is added to the network:

- sample (with influx and includes enzymes);
- blank (no influx, includes enzymes); and
- negative control (with influx, no enzymes).

Each sample has three replicates, constructed by initializing the network with different starting concentrations (a constant base concentration with random Gaussian noise). Each sample was “measured” at eight different time points. The ground truth of the dataset relates to whether the metabolites are in a network where the sample and negative control class receive an external influx (relevant metabolites) or not (irrelevant metabolites). The features originating from these networks—and only these—are the features of interest, further called the interesting or relevant features. In the training dataset, the number of relevant vs. irrelevant metabolites is balanced, i.e., there is an equal amount of each. There are 100 interesting features originating from networks one and three, and 100 uninteresting features originating from networks two and four (Figure 1). In the validation dataset, only one of twenty networks receives an influx (sample and negative control). In the other networks, there is no influx. This design results in 50 metabolites (those of the first network) that are affected because they are in a network that undergoes changes originating from external influxes. These and only these are the metabolites of interest. The other 950 metabolites are the uninteresting or irrelevant features. Effectively, this results in a ground truth where 5% of the 1000 metabolites are biologically relevant. These are the metabolites that the tools should discriminate from the remaining 95%.

The concentration flow in the network is governed by ordinary differential equations [13] that are solved numerically by using the standard Euler equations with a sufficiently small time step. More specific information can be found in Section S2 in Supplementary Materials along with a small example network to illustrate the mathematics of changes in the network over time. The parameters for simulating these networks are discussed as well as how to construct networks that have a connectivity distribution according to a power law [14]. All code, examples, and instructions on how to perform these simulations can be found in the MetaboLouise R package on github.com/Beirnaert/MetaboLouise and CRAN.

### 2.2. Statistical Methods for Differential Metabolism

The EDGE method [7,8], originating from genomics, is used to find the features that exhibit differential metabolism over time. The main differences between differential expression over time of genes and metabolites are the scaling and transformations that are applied to the data. Differential gene expression data are often log transformed because roughly half the data points are in the [0,1] range, with 1 signifying no difference in expression, and the other half in the range [1,∞]. By log transforming these data, the distribution changes to half of the data below 0 and half above 0. For liquid chromatography–mass spectrometry (LC-MS) or nuclear magnetic resonance (NMR) spectroscopy metabolomics data, this problem does not occur. The intensities values of these experiments are in the range [0,∞] (depending on NMR preprocessing steps negative values can occur). Therefore, only a linear transformation is applied so that each feature has a maximal intensity of 100 (divide by the maximum and multiply by 100).

**Figure 3:**
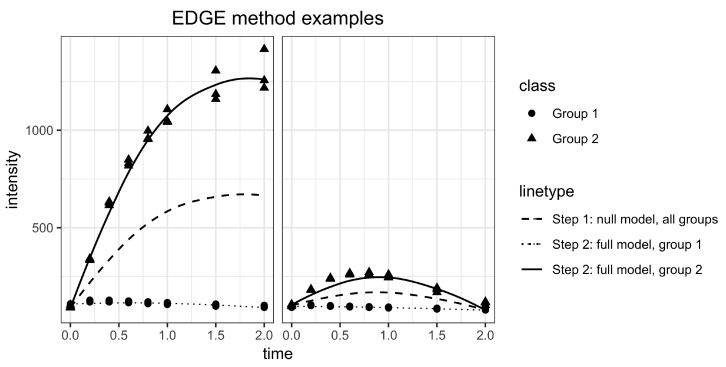
Illustration of the two steps in the EDGE algorithm for two different features (**left**, **right**). Step 1: The null hypothesis states that there is no difference between the groups. A single cubic spline curve is fitted to all data (dashed line). Step 2: In the alternative hypothesis, a curve is fitted to each group separately (dotted and full line). The improvement in goodness of fit is a measure for the difference between the groups.

EDGE works in two steps to compare whether two groups exhibit significant differential expression over time [7]. In the first step, the null hypothesis is formed stating that there is no difference between the two population time curves. A single natural cubic spline curve is fitted to all the data and the goodness of fit is calculated. Splines are chosen to keep the method flexible enough to capture different types of dynamics. In the second step, the alternative hypothesis is formed and a separate curve is fitted to each group (Figure 3). A likelihood ratio test is performed to obtain a *p*-value that quantifies the improvement in goodness of fit. A large improvement signifies more evidence for a difference in time profiles between classes. Note that *p*-value corrections such as false discovery rate (FDR) controlling procedures [15] are required in the case of multiple testing (Section S3 in Supplementary Materials). The EDGE algorithms contains a parameter to set the degrees of freedom for the curve fitting procedure. The default value is two, which is also the value used in the analysis. With two degrees of freedom the model is able to capture different dynamics without the risk of overfitting, as shown in Figure 3.

### 2.3. Machine Learning and Tinderesting

The aim is to use a machine learning (ML) model that emulates the human revision process. The reason for this is twofold. First, the removal of false positives from the results of a statistical analysis is a tedious manual labor, which can be supported by a trained machine learning model. Second, in the case of multiple biological sample classes in a single experiment, it can become non-trivial to interpret or combine the results of statistical methods. A random forest model was chosen as it outperformed a naive Bayes classifier and support vector machine (Section S4 in Supplementary Materials). The default parameters were used to train the random forest since the randomForest package uses appropriate defaults based on the data size [16] (Section S4 in Supplementary Materials). Note that, depending on the data, it can be beneficial to tune these parameters with nested cross-validation. Because parameter tuning is not common knowledge to many researchers and has the potential to overfit the data when it is not properly implemented, this falls beyond the scope of this work.

**Figure 4:**
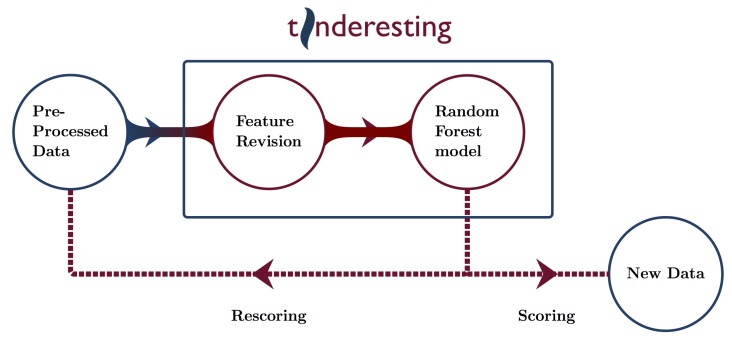
Overview of possible tinderesting workflows.

To train the ML model, labeled data are needed. These labeled data originate from the tinderesting Shiny app (Section S5 in Supplementary Materials), which is used by the experts to review the features. The experts have three options for labeling a feature: interesting, uninteresting or unknown. These correspond to the tinderesting labels. The unknown label indicates edge cases where the expert is uncertain. Features with the unknown label are omitted when constructing the final random forest model. Thus, the final model is built by using the features that have a tinderesting label that is interesting or not. These features serve as the samples for the machine learning model (every sample to classify corresponds to a metabolic feature rated by tinderesting) and the machine learning features are the individual elements of those metabolic features, i.e., an intensity for each sample class, replicate and time point combination. The performance is evaluated by receiver operator characteristic (ROC) with accompanying area under the curve (AUC) values as well as by precision–recall (PR) curves. Cross validation (10 fold) is used to obtain an unbiased estimate of the performance of the machine learning model (performance is evaluated on the hold-out fold) [17]. After the training procedure, the machine learning model can be used to score new data or rescore the entire dataset to look for potential false positives or false negatives in earlier statistical results (Figure 4).

In this study, the model was used to score the dynamic metabolomics validation dataset. Performance was evaluated by using ROC and PR curves. It is important to note that, when applying tinderesting for rescoring, care needs to be taken to avoid overfitting. Specifically, the features already used to train the tinderesting model need to be removed from the dataset on which to apply rescoring.

## 3. Results

### 3.1. Simulating Dynamic Metabolomics Data

**Figure 5:**
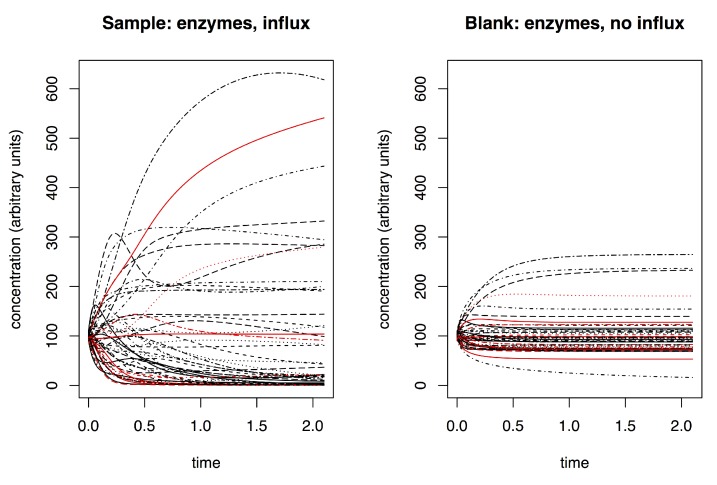
Example of time dynamics in a simulated metabolic network. (**left**) Example of an influx receiving network (between time 0.0 and 0.5); (**right**) the same network without external influx. Each line is a single metabolite (node in the network); a red line in the left plot indicates a metabolite (node) that receives an influx, whereas this metabolite does not receive an influx in the right plot. A single metabolic network underlies both plots. The network was initiated with the same starting parameters in each case (fixed starting concentration with Gaussian noise). The inclusion of an external influx results in different dynamics and in a different end state. Note that the units on the time axis correspond to the time units of the system of differential equations. In a way, these are arbitrary as the time scale of the dynamics depend on how the system is set up. Lower enzymatic concentrations will result in slower dynamics and vice versa.

**Figure 6:**
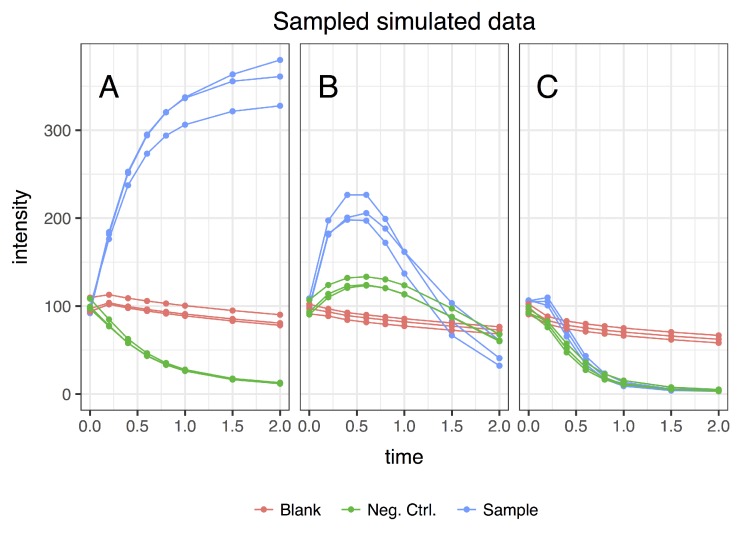
Three example features of the final simulated dataset. Taking the simulated time dynamics data in Figure 5, sampling these data and combining them by sample results in the final dataset to be used for EDGE and tinderesting. A single green line corresponds to the sampled data of a single continuous metabolite concentration signal (one line in the left of Figure 5). That same metabolite sampled in the right half of Figure 5 results in a single red line in this image. The three lines of each class are the replicates (i.e., same network, slightly different starting conditions because of noise). (**A**) A typical curve of metabolite formation; (**B**) an intermediary biotransformation; and (**C**) an uninteresting feature (sample class shows identical behavior as an other class).

The result of the dynamic metabolomics data simulation is a time curve of the concentration for each metabolite (Figure 5). These continuous ground truth data are sampled at discrete time points, corresponding to actual experiments where the underlying biological process is continuous but samples are taken only at distinct time intervals because of practical limitations (timely LC-MS runs, sample volume limitations, etc.). The resulting simulated data for a single metabolite are visualized in Figure 6. The dynamics in these simulated data correspond visually to example dynamics from actual experiments [10] (Section S6 in Supplementary Materials).

### 3.2. Statistical Analysis and tinderesting

**Figure 7:**
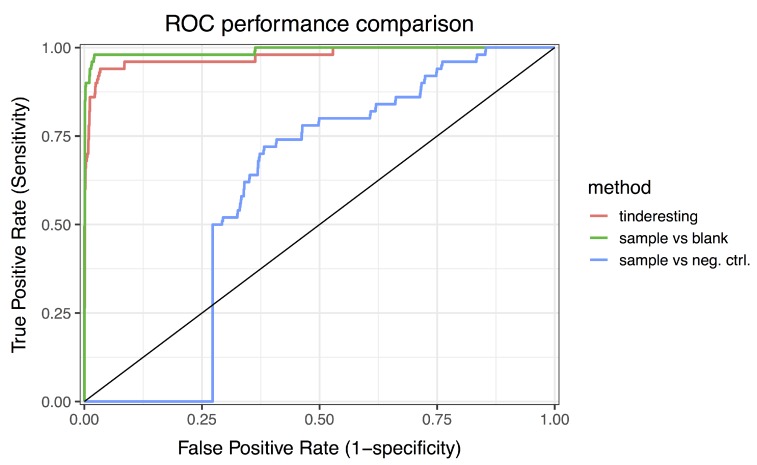
Receiver operator characteristic curves of tinderesting and EDGE. The area under the ROC curve (AUC) values are 0.977, 0.992 and 0.675 for tinderesting, sample vs. blank and sample vs. negative control, respectively.

**Figure 8:**
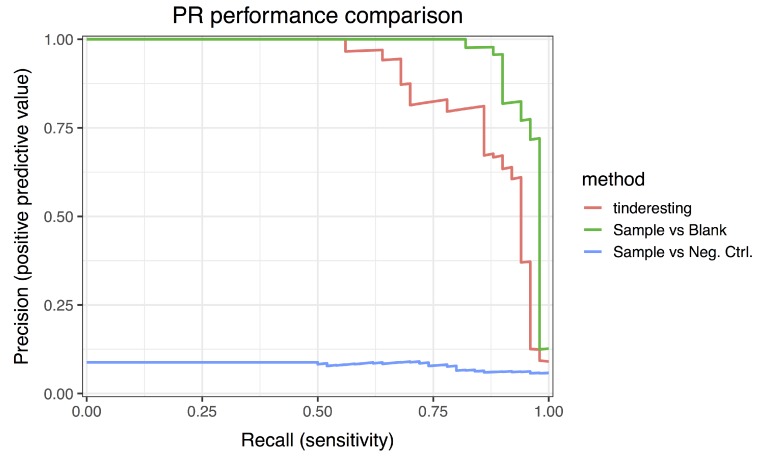
Precision–recall curves of tinderesting and EDGE. The precision–recall curves illustrate the same overall results as the ROC curves. This indicates that the performance is not due to class imbalance artifacts.

The statistical analysis was performed via the EDGE method [7,8]. For longitudinal metabolomics, this method is robust and performant for two class experiments, i.e., case vs. control. For example, the EDGE analysis for the difference between sample and blank results in an area under the ROC curve (AUC) value of 0.992 (Figure 7: ROC curve, Figure 8: precision–recall curve), which is a near perfect classification between biologically relevant and irrelevant metabolites. The difference between relevant and irrelevant is determined by which network they originate from (see Figure 2). In this case, only the metabolites in the first network are the biologically relevant ones as this network contains a number of nodes receiving an external concentration influx. For the comparison between the sample and negative control class, the EDGE performance is significantly lower (AUC 0.675, Figure 7 and Figure 8). When studying the experimental setup in Figure 2, it is clear that the optimal analysis would be to only look at the difference between sample and blank. However, in actual experiments, this may not be so clear and it is often the case that the experimental setup requires samples to be different from both blank and negative control samples. In this case, it is non-trivial to combine the two outcomes of an EDGE analysis. Simply combining the two would lead to suboptimal results; the sample vs. blank analysis should receive more weight in this specific example. Setting these weights is not straightforward. In addition, by combining the analysis, the number of statistical tests per feature is effectively doubled in this case. This theoretically leads to an increase in false positive results.

The number of evaluations in tinderesting is independent of the number of sample classes, circumventing the multiple testing issue, thus there are no difficulties in combining results. The outcome is a single score for each feature, independent of the number of samples, groups or classes that are present in the features. To avoid overfitting, the tinderesting model was trained on a different simulated dataset (Section S4 in Supplementary Materials). This dataset contained 200 samples, of which 77 were labeled interesting, 108 uninteresting and 15 samples for which the reviewer was uncertain. The uncertain samples were omitted resulting in the final training data consisting of 185 samples. Although the dataset is imbalanced (42% to 58%), it is reasonable to argue that this is not a major imbalance to warrant the need for sampling techniques such as SMOTE [18]. Using receiver-operator-characteristics and precision–recall curves, it is possible to measure the performance without being greatly affected by the imbalance.

**Figure 9:**
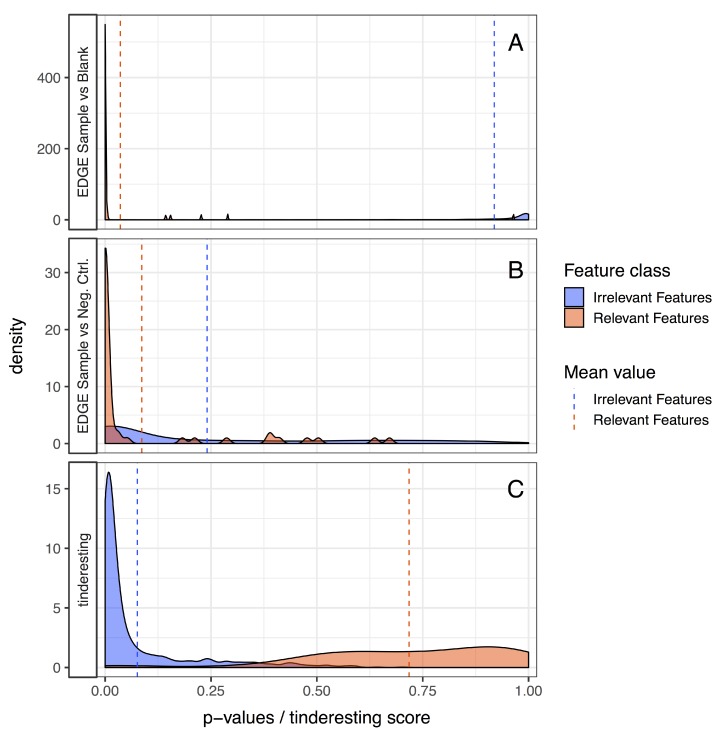
EDGE vs. tinderesting, comparing densities: (**A**) uncorrected EDGE *p*-values of sample vs. blank; (**B**) uncorrected EDGE *p*-values of sample vs. negative control; and (**C**) tinderesting scores. The relevant features originate from the network containing metabolites with a temporary external influx (50 features). The irrelevant features originate from the other networks (950 features). There is little overlap between the distributions of irrelevant and relevant features for the EDGE analysis of sample vs. blank and tinderesting case. The sample vs. negative control case shows a large overlap.

The training samples have 72 features each (8 time points per sample × 3 replicates per sample class × 3 classes). Cross validation was used to quantify the performance of the model on the training data (AUC 0.963, Section S4 in Supplementary Materials). This model was then used on the validation dataset, specifically to predict the probabilities for samples to belong to the interesting class. The random forest ROC curve (Figure 7) has an AUC value of 0.977, which is below the optimal EDGE score but not significantly different (Table 1). The precision–recall curves (Figure 8) illustrate similar results. Besides using performance metrics, the results can also be visualized via distributions (Figure 9, see also Section S7 in Supplementary Materials). The difference between relevant and irrelevant features is once again clear.

## 4. Discussion

To process the simulated dynamic metabolomics dataset with the tinderesting tool, 200 simulated features were reviewed based on their relevance. This information was used to train a random forest model to evaluate the simulated experiment whereby 50 metabolites (features) out of 1000 are relevant. The tinderesting model performed on par with the optimal statistical setup, which is often not known in actual experiments. The merit of this approach is to be found in the agnostic nature of the method with respect to the setup of the experiment. No matter the amount of sample classes or time points in the experiment, every feature will always receive a single score. This is in contrast to the case vs. control statistical tools which will produce a single score (usually a *p*-value) per feature for every two-class comparison. The one prerequisite is that the data can be readily visualized in a way that offers the expert a quick view on whether a feature is interesting or not. This is indeed the case in dynamic metabolomics experiments. The tinderesting method overall exhibited a slight decrease in performance compared to the statistical ideal situation; however, this is greatly offset by the advantages tinderesting offers in real life experiments with often complex designs. Peeters et al. [10] used tinderesting on experimental dynamic metabolomics data on the biotransformation of hederacoside C demonstrating the validity of the approach. In Section S8 in Supplementary Materials, an experimental dynamic metabolomics validation dataset is used to illustrate the training and performance evaluation procedure for experimental data. The experiment focusses on the gastrointestinal biotransformation [10,21] of quercetin, a flavonoid found commonly in plants [22].

The training data used in this validation case is relatively balanced. However, if one class is greatly overrepresented compared to the other, issues may arise when training machine learning models and evaluating their performance. When the result after expert revision with tinderesting is a dataset that contains very little interesting features and many uninteresting ones (or the other way around), it can be worthwhile to mitigate this situation. This can be done before the tinderesting step, for example by using EDGE upfront there can already be a first selection as to which features are possibly interesting. However, after the tinderesting revision process, it is also possible to account for the imbalance by using under-sampling or over-sampling. Methods such as SMOTE [18] over-sample the minority class to combat the imbalance.

A drawback of tinderesting is that the model is effectively trying to emulate the expert, which introduces a risk for bias. Hence, careful measures need to be taken to display the correct information to be reviewed by the expert. For example, information with regard to the identifications of features to be reviewed are irrelevant to the revision process. Including such information can cause a favorable bias towards the metabolites that the researcher wants to find (e.g., by spending more time reviewing these features). In addition, if multiple experts are included, it is beneficial to check for expert consistency. Although this has not been addressed in this paper it is possible with tinderesting, because the individual reviewers responses are labeled with an expert identifier. The calculation of majority votes could remove certain expert bias.

The method inherently carries the disadvantage that a new model needs to be trained for every experiment, or at least for every experiment with a different setup. If Experiment A is a metabolomics study where five time points are measured, it is impossible to use this experiment to train a model for an otherwise identical Experiment B that contains different time points. This is also true for experiments with different setups, different instruments, etc. The advantage, however, is that in experiments with a vast number of features, only a small part need to be reviewed for the model to work (200 for the model in this paper). This is a process that can be completed in a few minutes to maximally one hour. In addition, in experiments with a standardized processing pipeline, this method of feature revision can be used to evaluate the quality of the results. A model trained to filter out false positive results from true positive results can in turn be used to find false negative results, thus effectively allowing quality control on data processing pipelines.

In this paper, the dynamic metabolomics example of enzymatic prodrug metabolism was used to illustrate the approach. However, the broad applicability of machine learning allows the use of this technique for other pathways underlying the dynamic process, such as non-enzymatic reactions. The only requirement is that the dynamic needs to exhibit a pattern recognizable by experts. An additional interesting use case is the simulation of known metabolic pathways and the comparison to experimental data studying these pathways.

## 5. Conclusions

In this paper, we present the tinderesting tool to collect expert knowledge in an easy and quick manner via a Shiny app. The expert(s) rate(s) features or images as being interesting or not. To illustrate the method, we used it to analyze a simulated dynamic metabolomics dataset, which allows the use of a ground truth for performance evaluation (i.e., the relevant features are known). Although this dataset was constructed specifically for this paper, this method of simulating data can be relevant for other tools working with longitudinal/dynamic metabolomics data as the dataset is comparable to experimental longitudinal data [10].

The functions to generate the simulated dynamic metabolomics data are available in the MetaboLouise R package, available on github.com/Beirnaert/MetaboLouise and CRAN. A light version of tinderesting is also available on github.com/Beirnaert/tindeResting-template. The light version uses a folder with pre-generated images for the reviewing process. This allows the easy setup of the app without the need for significant adjustments in the app to obtain the correct plots. Possible interesting additions to the workflow can come from the active learning field. When the tinderesting app, after an initial cold start phase, can query the expert with cases that lie close to the model’s decision boundary, the learning rate can potentially be increased.

## Figures and Tables

**Table 1:** Statistical comparison of the multiple ROC curves and corresponding AUC values with the Venkatraman [19] and DeLong [20] tests, respectively.

Tinderesting AUC	EDGE AUC	ROC Test *p*-Value	AUC Test *p*-Value
0.977	0.675	< 0.001	< 0.001
0.977	0.992	0.026	0.165

## Supplementary Materials

The following are available online at https://www.mdpi.com/2218-1989/9/3/54/s1, Figure S1: Generated biological network connectivity distribution, Table S1: Simulation parameters, Figure S2: Metabolomic network example, Figure S3: ROC curves for random forest, SVM and naive Bayes classifiers, Figure S4: Precision-Recall curve for the random forest classifier, Figure S5: Visualization of the full tinderesting app, Figure S6: Two examples from a dynamic metabolomics experiment, Figure S7: Feature scores of EDGE and tinderesting, Table S2: XCMS pre-processing parameter settings, Figure S8: ROC curve for tinderesting on expert-reviewed experimental data.
