# Supplementary material for: Using Expert Driven Machine Learning to Enhance Dynamic Metabolomics Data Analysis
Source: Metabolites. 2019 Mar 20;9(3):54. doi: 10.3390/metabo9030054 (PMC6468718; doi:10.3390/metabo9030054)
Supplement: Supplementary file 1 [file metabolites-09-00054-s001.pdf]

Supplementary Materials:

S1: Simulating biologically relevant metabolic networks

An important factor in generating random biological networks is the distribution of the connectivity. Biological networks follow a power law distribution [1], therefore it is important to maintain this property in the simulated networks. The Barabási-Albert model [2] allows the construction of networks with the appropriate distribution (Fig S1). Note that it is important to inspect the network connectivity matrix for the following properties:

1. asymmetry: the network matrix cannot be symmetric with respect to the main diagonal, this would imply that every two metabolites that are connected, are connected in both directions. This is not the case in a biological network.
2. non-triangularity: if the network matrix exhibits a triangular shape (lower or upper triangularity), then certain nodes (metabolites) have a larger probability of having many connections. When simulating many networks this would result in, for example, metabolite A having the most connections in most of the simulations. This is non beneficial to the randomness of the generated metabolic networks. If a triangular matrix has the correct power-law distribution, the triangularity problem can be solved by randomly shuffling columns or rows.

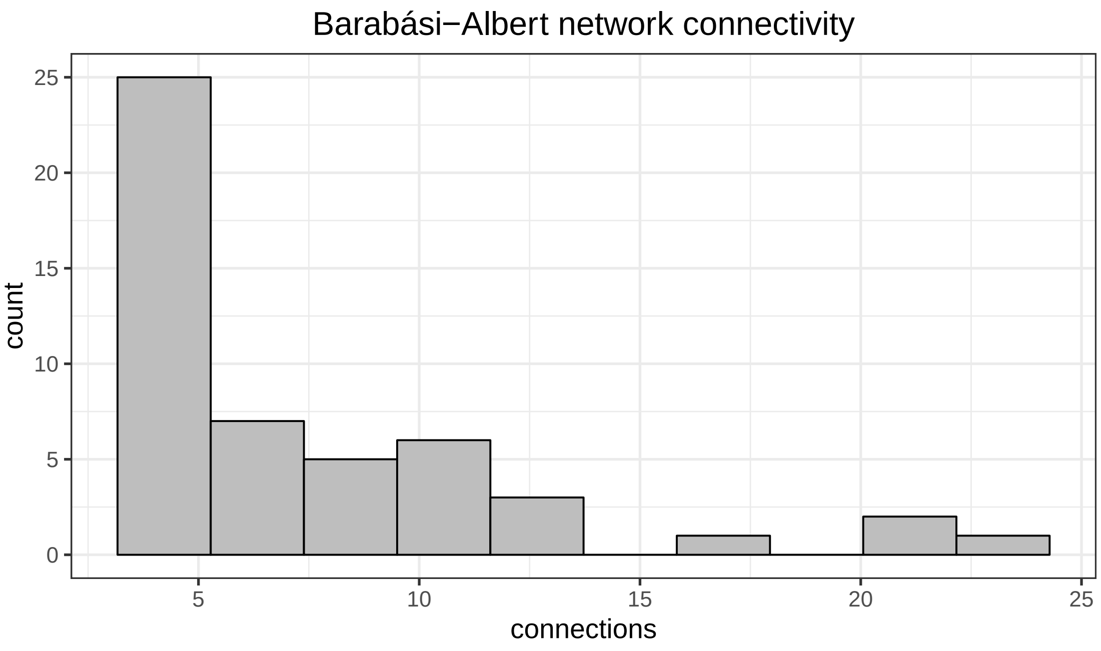

Figure S1. Generated biological network connectivity. The network generated with the Barabási-Albert algorithm exhibits a connectivity distribution according to a power law.

Table S1. Simulation parameters.

Parameter	Value	Description
Nreplicates	3	The number of replicates for each sample. For each replicate the starting concentrations are different but the network is the same.
Nmetabos	50	The number of metabolites/nodes in the network
dt	0.001	The time step (taken sufficiently small for the Euler approximation to hold).
tmax	2.1	The end time.
start_concentration	100	The starting concentration of each metabolite without noise.
max_abs_concentration_noise	10	The maximal absolute value of the noise that is added to starting concentration. Set at 1/10th of start_concentration.
influx_tmax	0.5	The time point at which the influx (if present) stops.
N_influx	10	The number of metabolites that receive an influx.
Neg_ctrl_protein_factor_strong	0.01	The factor by which the rates are multiplied in the case of a "no influx, no enzymes" simulation (Fig **??**).
Neg_ctrl_protein_factor_weak	0.5	The factor by which the rates are multiplied in the case of a "no influx, little enzymes" simulation (Fig **??**).
Neg_ctrl_protein_fraction	0.5	The fraction of enzymes that receive the strong negative control protein factor.
BApower	0.5	Parameter of the Barabási-Albert graph model generator from the igraph R package [3]: The power of the preferential attachment
BA_mValue	4	Parameter of the Barabási-Albert graph model generator from the igraph R package: the number of edges to add in each time step

S2: Dynamics of a metabolic network, the mathematics of change over time

For the example the following small network [1] is used:

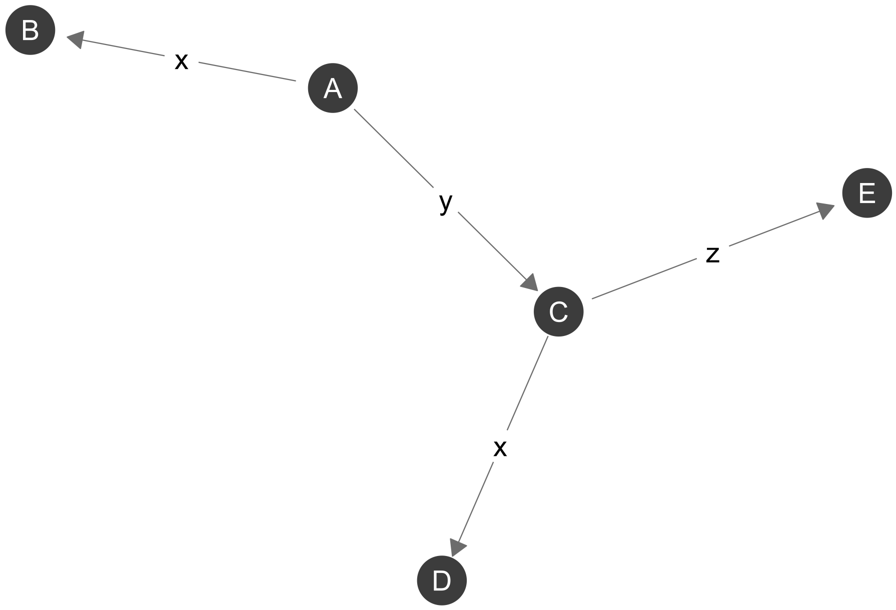

Figure S2. Metabolomic network example. Nodes are metabolites and the edges represent flow between metabolites. These flows are rates based on concentrations of enzymes.

The nodes represent metabolites, A, B, C, etc. that have certain concentrations denoted as A, B, C respectively. These concentrations evolve based on the enzymes x, y and z. These are effectively the rates that govern the flow and are denoted as x, y and z respectively. Thus, the evolution of the concentration over time can be written according to the following ordinary differential equations:

$$\begin{aligned}\frac{\mathrm{d}A}{\mathrm{d}t} &= -(x+y)A \\ \frac{\mathrm{d}B}{\mathrm{d}t} &= xA \\ \frac{\mathrm{d}C}{\mathrm{d}t} &= yA - (x+z)C \\ &\vdots\end{aligned}$$

To numerically solve these equations the Euler approximation is used by substituting the following finite difference scheme into the equations for the metabolic network

$$\frac{\mathrm{d}f}{\mathrm{d}t} \approx \frac{f(t+\delta t) - f(t)}{\delta t}$$

by writing $f(t+\delta t)$ in the following shorthand notation $f_{t+\delta t}$ we obtain the following

[1] Visualized with the ggnet R package.

$$\frac{A_{t+\delta t} - A_t}{\delta t} = -(x+y)A_t$$
$$\frac{B_{t+\delta t} - B_t}{\delta t} = xA_t$$
$$\frac{C_{t+\delta t} - C_t}{\delta t} = yA_t - (x+z)C_t$$
$$\vdots$$

Reworking this gives the final result for the state of the network at time point $t + \delta t$ in function of the state at time point t

$$A_{t+\delta t} = -\delta t(x+y)A_t + A_t$$
$$B_{t+\delta t} = \delta t x A_t + B_t$$
$$C_{t+\delta t} = \delta t(yA_t - (x+z)C_t) + C_t$$
$$\vdots$$

When choosing δt sufficiently small the Euler approximation is valid. As mentioned in the manuscript, the rates are influenced by the concentration of metabolites. Specifically, when the concentration of a metabolite increases, the rates that deplete that metabolite will also increase, up to a certain maximum. The sigmoid function is suited to this behavior, for example, the equation governing the evolution of rate y is

$$y(A_t) = \frac{y^{max}}{1 + \exp[-k(A_t - A_0 - c)]}$$

with c a parameter that needs to be set according to the following equation

$$c = \frac{\ln(y^{max} - 1)}{k}$$

This parameter shifts the sigmoid function so that the value is 1 at the starting concentration y^{max}.

S3: Multiple testing correction problem for EDGE and metabolomics

A critical note on p-values and multiple testing correction has to be made. EDGE performs a test for an improvement in model performance. This is effectively a one-sided test i.e. the full model with two curves performs better than the null model with 1 curve. Large numbers of these one-sided tests can result in a bimodal p-value distribution. Such a bimodal p-value distribution can prevent the adequate application of the often used FDR correction, as it uses the distribution of the p-values to estimate the π_0 value (proportion of true nulls), and this distribution is assumed to be roughly uniform in the high p-value region [5]. With regard to bimodal p-values, such a distribution can further be caused by a large number of features without any difference between sample classes, This can often occur in biological experiments as large numbers of (uninformative) features are measured. In this case, the bimodal distribution problem can be solved by either removing these features or by applying surrogate variable analysis techniques, which can also be used to remove batch effects and other unwanted variation.

S4: Performance comparison of machine learning models.

To find the optimal machine learning model for the longitudinal metabolomics data the predictive performance was evaluated on the external tinderesting data as discussed in the main manuscript. The compared models are:

1. a random forest with 500 trees, 8 variables sampled at each split, minimum size of terminal node 1, sampling with replacement (the default parameters from the randomForest R package [6]),
2. a support vector machine with a radial basis kernel (rest of parameters are default from e1071 R package)
3. a naive Bayes classifier (default e1071 R package parameters, no priors set).

The performance of the three classifiers was compared in a 10 fold cross validation setup, each repeated 20 times with different folds (to get an estimate of the ROC curve variability). The results are visualized in Fig S3. The random forest model consistently outperforms the SVM and naive Bayes classifier[2]. The code to run these models and obtain similar plots is available in the MetaboMeeseeks R package [7]. For completeness, the Precison-Recall plot of the Random forest model is plotted in Fig S4. The random forest model trained with this simulated data is used to compare to the EDGE model. This is to avoid overfitting on the data and to allow a fair comparison between EDGE and tinderesting.

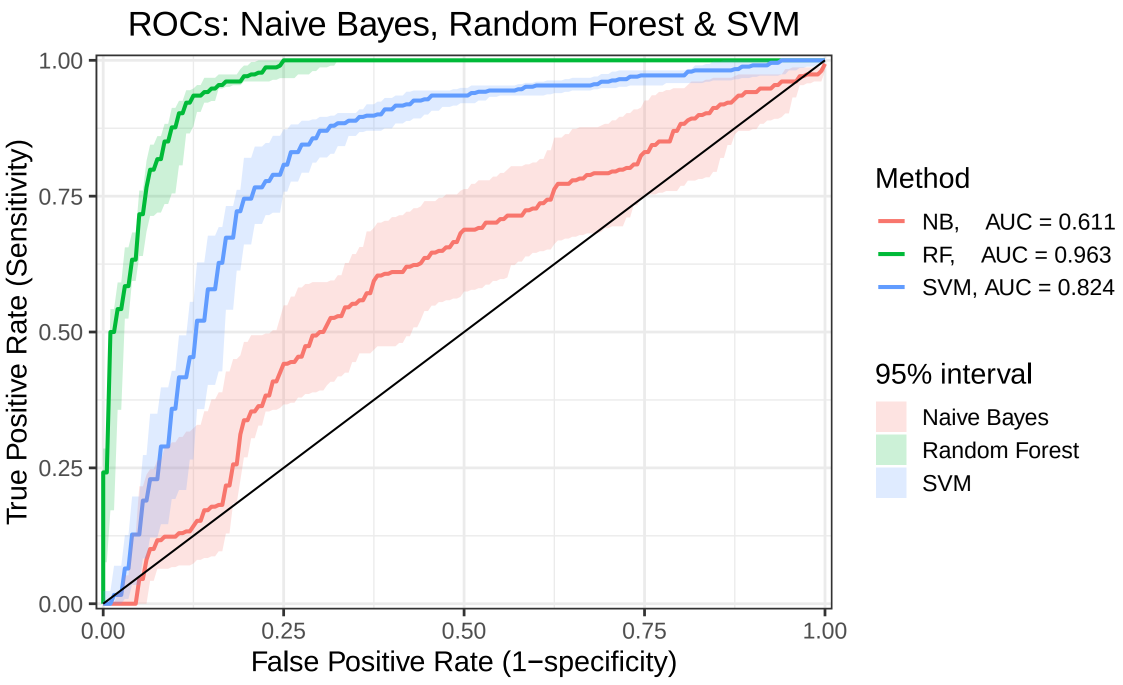

Figure S3. ROC curves for random forest, SVM and naive Bayes classifiers. Classifier performance is represented with the respective receiver-operator-characteristics. Comparing the area under the ROC curves (AUC values) justifies the choice for the random forest classifier.

[2] The SVM and naive Bayes model are implemented with the e1071 R package.

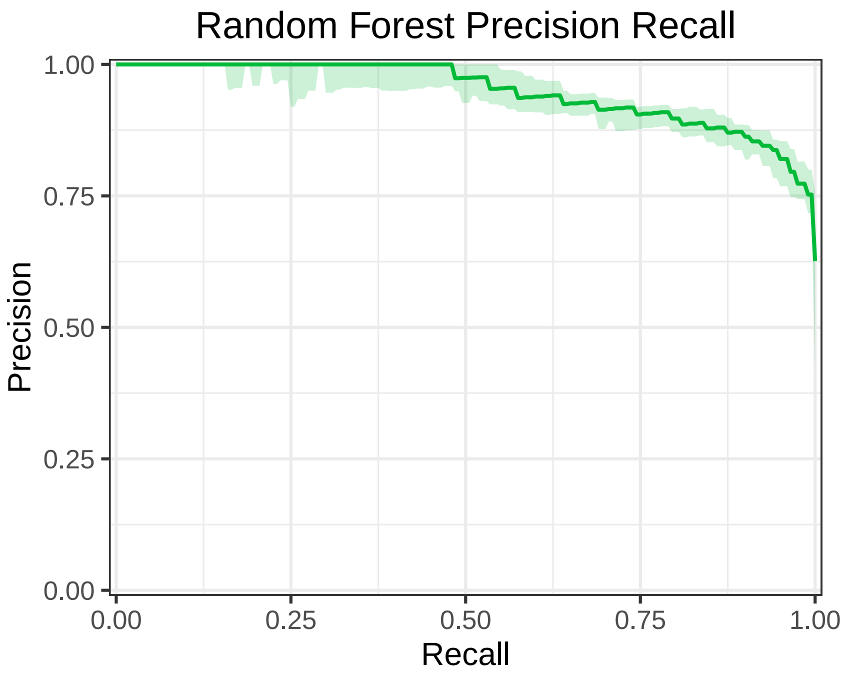

Figure S4. Precision-Recall curve for the random forest classifier.

S5: Shiny app and user interaction

To review the quality of the significant features and to train the machine learning model, we constructed a Shiny web app called tinderesting. Shiny is an R package to produce such interactive web apps that require little effort to construct and can easily be run on a server. The app queries the user for the quality of features, and uses these results to build a model in the background (Fig S5). All responses are stored in an SQLite database for straightforward storage and access.

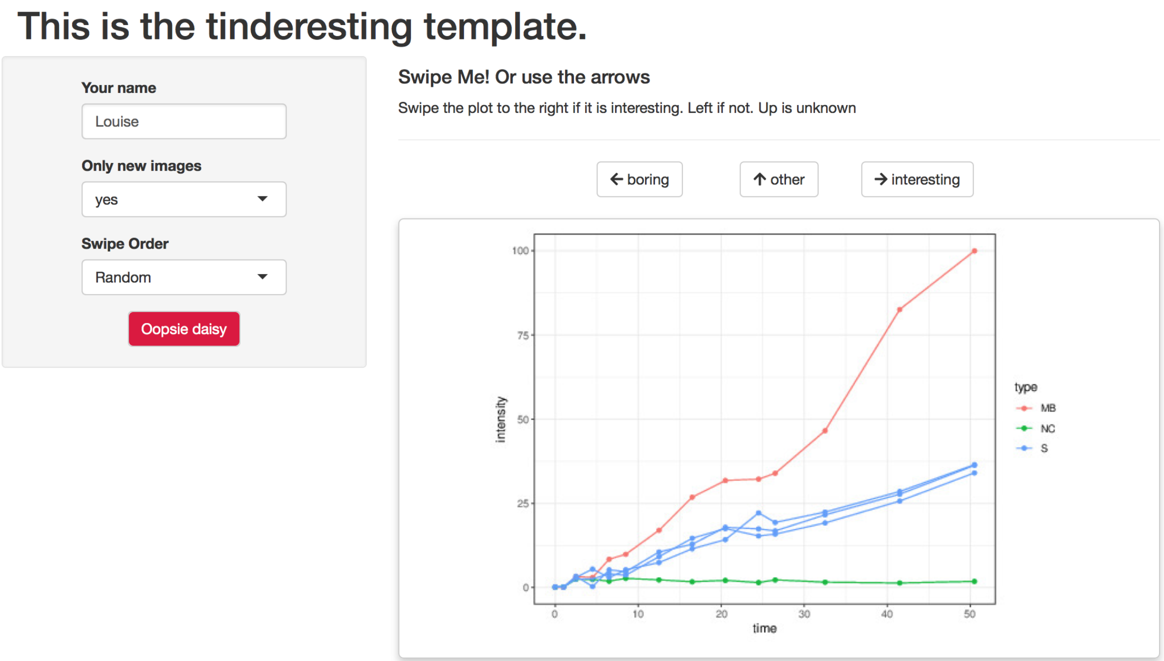

Figure S5. Visualization of the full tinderesting app. The user selects the subset of the data on the left hand side, the selected subset is visualized. Next, features appear on the right hand side that need reviewing by the expert. The results are logged at the bottom of the app and simultaneously stored in an SQL database.

69 **S6: Examples of actual dynamic metabolomics data**

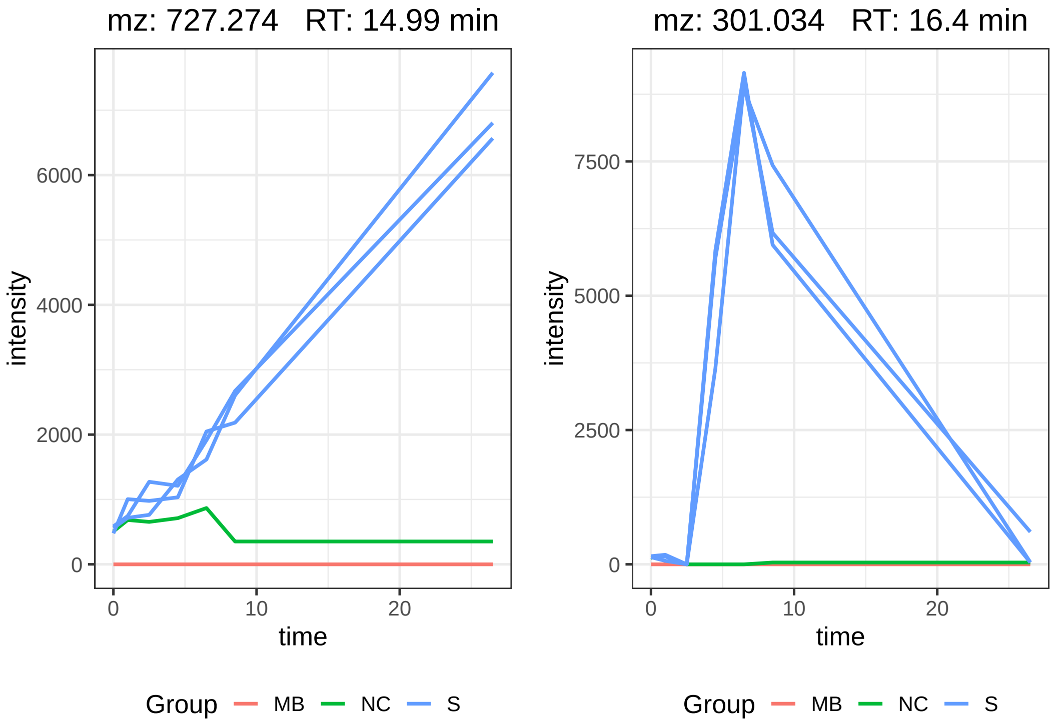

Figure S6. Two examples from a dynamic metabolomics experiment. Visualization of two features from the processed LC-MS dataset [4]. The sample class (S) shows different behavior over time compared to the other classes (MB = method blank, NC = negative control). Left, a biotransformation product is formed in the sample class and right, a biotransformation product is initially formed, followed by a further biotransformation.

S7: Extra visualization of tinderesting results

The vizualization in Fig S7 does not fully grasp the results as many features are plotted on top of each other, for example in the bottom left corner of the right plot. A dot is depicted for each irrelevant feature (left) or relevant feature (right). The colour of this dot represents the tinderesting score and the uncorrected p-values from EDGE are plotted on the x and y positions: sample (S) vs negative control (NC) and sample (S) vs blank (B) respectively. Overall, uninteresting features have low tinderesting scores and interesting ones have high scores. Also note that the irrelevant features are spread out over the y axis as the EDGE model for sample vs negative control model underperforms overall. Most of the relevant features are plotted on top of each other making it difficult to observe the actual tinderesting score

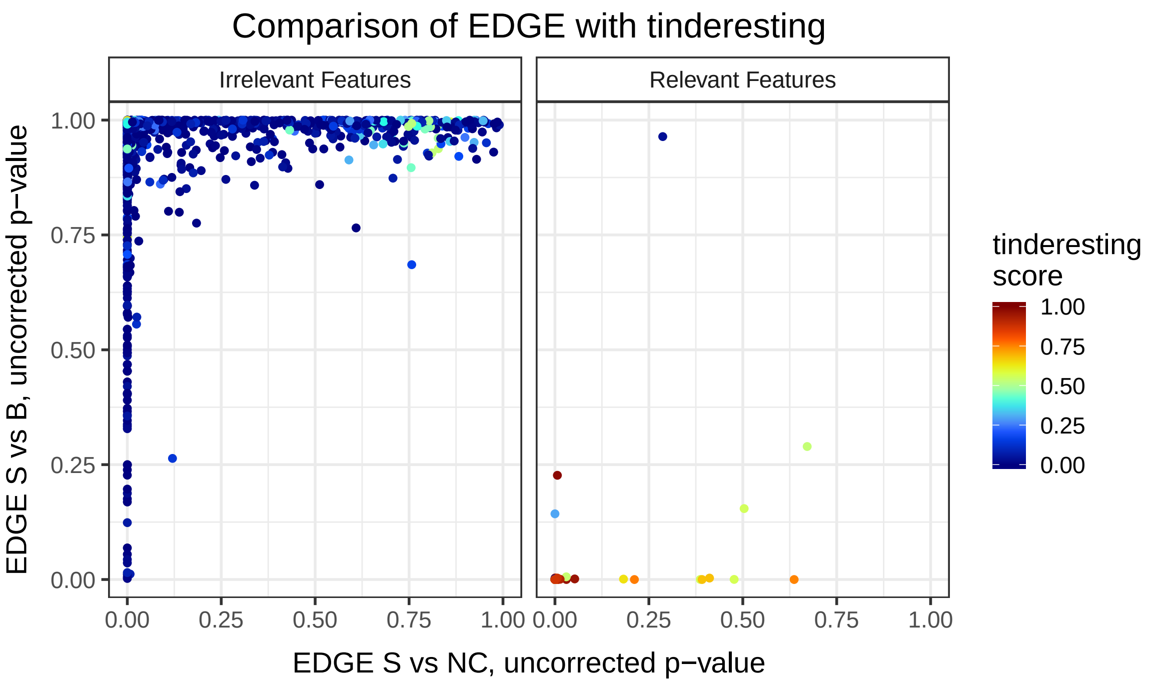

Figure S7. Feature scores of EDGE and tinderesting. Overall, the irrelevant features have a low tinderesting score (blue colour), and the relevant ones generally have a high score (red). The EDGE comparison of sample vs blank exhibits mostly very low (uncorrected) p-values for the relevant features. The (uncorrected) p-values of the EDGE sample vs negative control comparison are more spread out, which is consistent with the results of the performance comparison.

S8: Experimental validation dataset

A gastrointestinal model developed by Breynaert et al. [8] and adapted by Peeters et al. [4] was used to study the biotransformation of quercetin, one of the most abundant flavonoids found in plants. Details of the gastrointestinal model can be found in Peeters et al [4] including the experimental setup. Three sample classes were biotransformed in the gastrointestinal model consisting of stomach, small intestine and colon: sample, negative control and blank. The sample class contains quercetin and gut bacteria for biotransformation of the compounds were added during the colon phase. The negative control class contains the quercetin compounds but no bacteria. The blank sample contains bacteria but no quercetin. The objective is to find the metabolites that form after biotransformation of quercetin. Specifically, the time profile of the sample class should be different from both blank and negative control.

The experiments were performed on a Waters Xevo G2-XS QTOF instrument (accurate mass). Specific liquid chromatography and mass spectrometry settings and other experimental details can be found in Peeters et al. [4] as the exact same settings were used to for this quercetin experiment. Because the Waters Lockspray^{TM} was used in the experiment, the files were converted to the open source mzXML format with the msConvert tool from ProteoWizard [9]. Because the Waters lockspray technique was used, the method of Stanstrup et al. [10] was used to remove the lockspray runs. Next XCMS [11] was used for pre-processing the open source data files to obtain a matrix ready for further data analysis. Specifically the centWave function was used for peak-picking followed by grouping with the density method and finally peak-filling, see Table S2 for the parameter settings. The final data matrix contained 70 samples and 17793 features.

Table S2. XCMS pre-processing parameter settings

function	parameter	value
xcmsSet	method	"centWave"
	ppm	10
	peakwidth	c(5,25)
	mzdiff	0.01
	prefilter	c(3,5000)
	integrate	1
	snthresh	10
	noise	1000
group	method	"density"
	bw	5
	mzwid	0.015
	minfrac	0.20
	max	100
	minsamp	2

After the pre-processing steps an initial EDGE analysis was performed to find a subset of the data for expert revision. This step is not strictly necessary but it aides in reducing the amount of irrelevant features to be reviewed by the expert. Usually there will be a class imbalance towards the uninteresting features. An initial EDGE filtering can reduce this imbalance. The p-value thresholds for both EDGE analysis (sample vs blank and sample vs negative control) was set at 0.25. This resulted in 507 out of 17793 features to be reviewed by the expert. This resulted in 423 uninteresting features, 79 interesting features and 5 for which the reviewer was uncertain. Although an initial EDGE analysis was performed there is still a majority of uninteresting features. We can now use these expert-rated data as a validation dataset for the overall tinderesting approach. In Fig S8 the performance of tinderesting is illustrated by performing 10-fold cross validation on the expert-rated dataset. In each cross validated loop a random forest model is trained with the informed default parameters of the randomForest R

package [12]. The performance indicates that the model is capable to reproduce the expert labelling in most cases. Although the performance is lower than for the simulated dynamic data case but this is not unrealistic because experimental data is more diverse than simulated data.

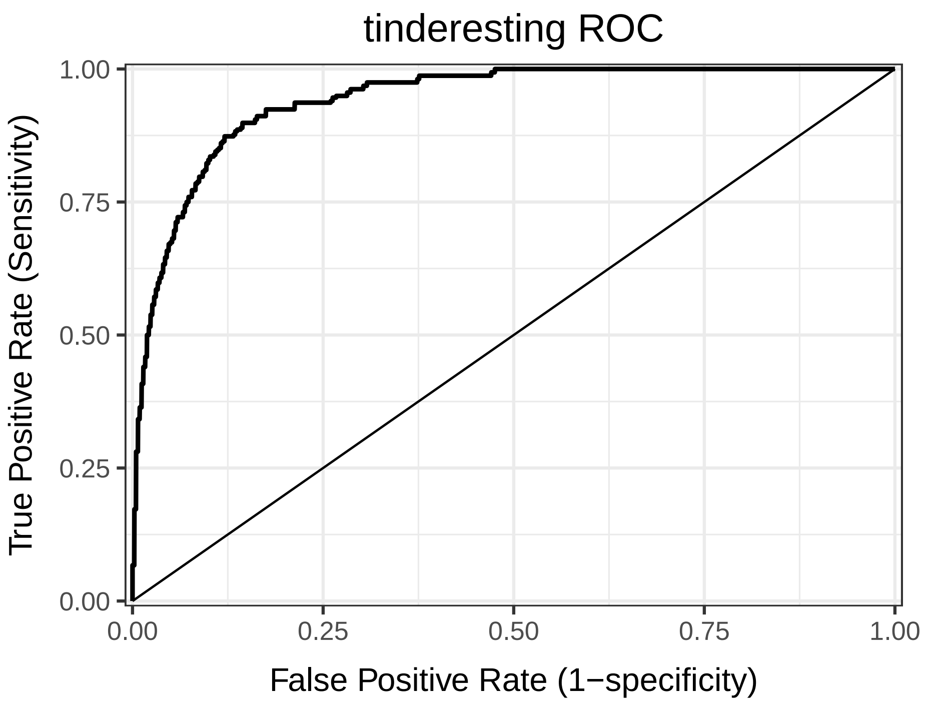

Figure S8. ROC curve for tinderesting on expert-reviewed experimental data.

Supporting references

1. Jeong H.; Tombor B.; Albert R.; Oltvai Z.N.; Barabási A.L.; The large-scale organization of metabolic networks. *Nature* **2000**, *407(6804)*, 651–654
2. Barabási A.L.; Albert A.; Emergence of scaling in random networks. *Science* **1999**, *286(5439)*, 509–512
3. Csardi G.; Nepusz T.; The igraph software package for complex network research. *InterJournal, Complex Systems* **2006**, *1695(5)*, 1–9.
4. Peeters L.; Beirnaert C.; Van der Auwera A.; Bijttebier S.; Laukens K.; Pieters L.; Hermans N.; Foubert K.; Revelation of the metabolic pathway of Hederacoside C using an innovative data analysis strategy for dynamic multiclass biotransformation experiments. *Journal of Chromatography A* **2019**, *in Press*.
5. Storey J.D.; Tibshirani R.; Statistical significance for genomewide studies. *Proceedings of the National Academy of Sciences* **2003**, *100(16)*, 9440–9445.
6. Liaw A.; Wiener M.; Classification and Regression by randomForest. *R News* **2002**, *2(3)*, 18–22
7. Beirnaert C.; Cuyckx M.; Bijttebier S.; MetaboMeeseeks: Helper functions for metabolomics analysis. *R package version 0.1.2* https://github.com/Beirnaert/MetaboMeeseeks
8. Breynaert A; Bosscher D.; Kahnt A.; Claeys M.; Cos P.; Pieters L.; Hermans N.; Development and validation of an in vitro experimental gastrointestinal dialysis model with colon phase to study the availability and colonic metabolisation of polyphenolic compounds. *Planta medica* **2015**, *81(12/13)*, 1075–1083.
9. Kessner D.; Chambers M.; Burke R.; Agus D.; Mallick P.; ProteoWizard: open source software for rapid proteomics tools development. *Bioinformatics* **2008**, *24(21)*, 2534–2536.
10. Stanstrup J.; Gerlich M.; Dragsted L.O.; Neumann S.; Metabolite profiling and beyond: approaches for the rapid processing and annotation of human blood serum mass spectrometry data. *Analytical and bioanalytical chemistry* **2013**, *405(15)*, 5037–5048.
11. Smith C.A.; Want E.J.; O'Maille G.; Abagyan R.; Siuzdak G.; XCMS: processing mass spectrometry data for metabolite profiling using nonlinear peak alignment, matching, and identification. *Analytical chemistry* **2006**, *78(3)*, 779–787.
12. Liaw A.; Wiener M.; Classification and regression by randomForest. *R news* **2002**, *2(3)*, 18–22.
